# A hot-electron thermophotonic solar cell demonstrated by thermal up-conversion of sub-bandgap photons

**DOI:** 10.1038/ncomms9685

**Published:** 2015-11-06

**Authors:** Daniel J. Farrell, Hassanet Sodabanlu, Yunpeng Wang, Masakazu Sugiyama, Yoshitaka Okada

**Affiliations:** 1 Research Centre for Advanced Science and Technology (RCAST), The University of Tokyo, 4-6-1 Komaba, Meguro-ku, Tokyo 153-8504, Japan; 2 Department of Electrical Engineering and Information Systems, Graduate School of Engineering, The University of Tokyo, 2-11-16 Yayoi, Bunkyo-ku, Tokyo 113-0032, Japan

## Abstract

The direct conversion of solar energy to electricity can be broadly separated into two main categories: photovoltaics and thermal photovoltaics, where the former utilizes gradients in electrical potential and the latter thermal gradients. Conventional thermal photovoltaics has a high theoretical efficiency limit (84%) but in practice cannot be easily miniaturized and is limited by the engineering challenges of sustaining large (>1,000 K) temperature gradients. Here we show a hot-carrier-based thermophotonic solar cell, which combines the compact nature of photovoltaic devices with the potential to reach the high-efficiency regime of thermal photovoltaics. In the device, a thermal gradient of 500 K is established by hot electrons, under Stokes illumination, rather than by raising the temperature of the material itself. Under anti-Stokes (sub-bandgap) illumination we observe a thermal gradient of ∼20 K, which is maintained by steady-state Auger heating of carriers and corresponds to a internal thermal up-conversion efficiency of 30% between the collector and solar cell.

## Abstract (web summary)

The efficiency of single junction solar cells is limited by their inability to absorb photons with an energy smaller than the bandgap. Here, the authors surpass the Shockley-Queisser limit by using an integrated hot-electron thermophotonic emitter to thermally up-convert sub-bandgap photons.

## Graphical abstract

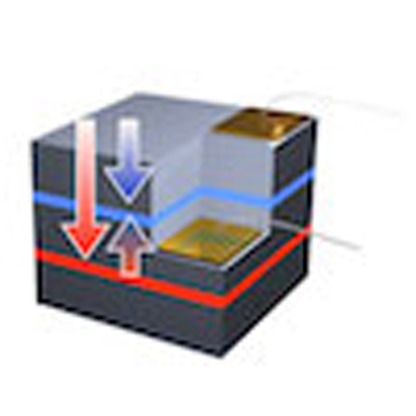

Single junction solar cells are limited to efficiencies of <31% (the well-known Shockley–Queisser limit) by the inability to absorb below-bandgap photons and the thermalization of photogenerated carriers to the band edges^1^. Thermophotovoltaic (TPV) energy converters on the other hand have a much higher limiting efficiency of 85% (ref. 2). In TPV, concentrated sunlight heats an external solar collector, which emits a broad thermal spectrum towards optimized low-bandgap solar cells. An interesting conceptual extension of TPV is thermophotonics (TPX), in which the emission rate between the solar collector and the solar cell is enhanced by the presence of both an electrochemical potential and a thermal gradient in the collector^3^,^4^. Here we demonstrate a thermophotonic device in which the thermal gradient is maintained by hot electrons in a quantum-well-based solar collector that is directly integrated into the device structure. Light emission from the hot electrons in the quantum wells provides additional optical power to the solar cell and thus boosts its efficiency.

The traditional ‘single-source' configuration for a TPV or TPX device consists of a thermal collector facing the Sun, followed by filters and then a solar cell. The filters prevent any below-bandgap light from entering the solar cell and reflect it back towards the thermal collector where it can be reabsorbed.

Here we consider an alternative ‘two-source' configuration in which the solar collector is located behind the solar cell (Fig. 1a,b). In this configuration the solar cell plays the role of the filter: sunlight illuminates the solar cell directly, below-bandgap light is transmitted and raises the temperature of the thermal collector located at the rear. This configuration relies on thermal up-conversion in the solar collector to increase carrier energy such that some radiative recombination will occur with energy above the bandgap of the solar cell (the blue region in Fig. 1a) and thus contribute to additional photocurrent generation. In this way the solar collector becomes the second source of photons and the device concept can achieve an efficiency above that of the Shockley–Queisser limit^5^.

Using hot electrons for light emission means that the optical behaviour is identical to conventional thermal collectors, but without the engineering challenges of maintaining a large temperature gradient (1,000 K) in close proximity to a temperature-sensitive solar cell. In our device, the lattice temperature of the solar cell and thermal collector remain at room temperature at all times—only the electrons in the collector are hot.

Integrating the collector with the solar cell has several practical and fundamental advantages. The all-solid-state approach has a simple flat plate form factor allowing the solar absorber layer to be added in a single growth process. Also, the collector emits into the internal optical modes of the solar cell, thus enabling a *n*^2^∼12 × enhancement above the radiative transfer rate for an externally located collector (this is the reason for the discontinuous spectrum shown in Fig. 1a).

The main challenge of hot-carrier approaches is the rapid electronic cooling that occurs in semiconductor absorbers. However, recently some remarkable demonstrations of slowed electron cooling in nanostructures have been published. Pandey *et al.*^6^ showed a specially designed colloidal nanostructure with an excited state electron lifetime extended from 1 ps to 1 ns. A number of experiments with multi-quantum wells (MQWs) have revealed anomalously slow ensemble cooling rate of 5 ns and also the ability to maintain very large electronic-lattice temperature gradients; carrier temperatures of 600 K have been recently reported^7^,^8^; here we report temperatures slightly below 800 K.

In the following, we highlight the opportunity to use hot carriers as novel spectral converters. This approach differs from that of the hot-carrier solar cell: it is simpler, it removes the requirement for hot-carrier transport and energy selective extraction, and can capitalize on recent developments in nanophotonics to tune absorption and emission characteristics. The main results we present are Stokes, anti-Stokes and photocurrent power-dependence measurements of a solar cell with an integrated hot-carrier solar collector. Under Stokes illumination we show very large hot-carrier temperature gradients of ∼500 K can be maintained. Under anti-Stokes illumination we show a modest temperature gradient (∼20 K) can be maintained and by analysing the power-dependence postulate that this is caused by Auger heating of carriers. The photocurrent results show a non-linear power dependence from entering the themophotonic operating regime.

## Results

### Experimental set-up

In the experiments that follow, the two-source configuration has been chosen to explore the thermophotonic properties of the solar collector. This is because a high intensity infrared (IR) laser (which is below the bandgap of the solar cell) can be used to simulate the truncated spectrum received by the solar collector. The usefulness of the two-source configuration is that any photocurrent recorded by the solar cell can be directly correlated to hot-carrier light emission received from the solar collector. This configuration prevents a situation in which direct generation of carriers in the solar cell could mask the signal originating from the thermal up-conversion process.

In principle a similar single-source configuration, in which the solar cell is placed behind the solar collector, is also possible with this scheme. However it would require a very high reflectivity filter (possibly a dielectric stack) to also be integrated into the device structure. In addition the filter's angular response would be critically important because of the angular spread of focused laser light. We believe that for the first set of experiments in this area the two-source configuration provides a simpler route to explore this concept. In addition it will also allow the role of thermal up-conversion and the efficiency of the process to be quantified experimentally.

For Stokes and anti-Stokes measurements a (50 mW) 808-nm laser diode and (500 mW) 1,064-nm diode-pumped solid-state (DPSS) laser are used, respectively. The 808-nm diode allows hot carriers to be generated directly in the MQWs and from these measurements the cooling coefficient of hot carriers can be measured. However, experiments with the 1,064-nm laser do not directly generate hot carriers, instead electrons and holes are directly generated in the ground state of the MQW absorber. Therefore, any observed increase in carrier temperature must originate from a self-heating mechanism: one possible mechanism is Auger recombination. Würfel has previously established the importance of Auger recombination and impact ionization in reaching equilibrium between a hot electron–hole plasma^9^. Auger recombination reduces particle number in the solar collector but allows the average energy per particle (the carrier temperature) to increase because the recombining electron and hole transfer their energy back into the electronic system by scattering a third electron (or hole) into a high-lying energy state.

The device structures considered here allow the temperature of Auger-heated carriers to be measured. Furthermore, because the integrated solar cell becomes a very sensitive photodetector, for light emitted by the solar collector, the total up-conversion efficiency can be directly measured.

### Thermodynamic efficiency limits

The thermodynamic efficiency limits of TPV and TPX are well-known^2^,^4^ and the thermodynamics of thermal up-converters have also been investigated^10^. Recently we presented the thermodynamic efficiency limits for single- and two-source TPV-like devices with integrated hot-electron solar collectors and also suggested how efficiency can be improved by controlling optical modes^5^.

The red region in Fig. 2a shows the boost in efficiency achievable with the two-source configuration. Here the bandgaps of the solar cell, solar collector and also the applied voltage have been globally optimized. For comparison, the blue region in Fig. 2a shows the globally optimize Shockley–Queisser limit as a function of solar concentration factor.

The model in ref. 5 is used to estimate the up-conversion efficiency (*EQE*_up_=*N*_up_/*N*_in_) occurring in the two-source configuration under different simulated illumination conditions, where *N*_up_ is the up-converted photon flux entering the solar cell and *N*_in_ is the total entering photon flux over all wavelengths. Figure 2b shows the predicted up-conversion efficiency of the device concept. The solar cell bandgap is fixed at 1,000 nm (1.24 eV) and the value of the solar collector placed behind the solar cell is changed. The dashed vertical line corresponds to the bandgap combination used in this study.

The blue region in Fig. 2b shows the upper limit of *EQE*_up_ when the solar cell bandgap is fixed to the value used here (1,000 nm) and the device is illuminated with fully concentrated sunlight. This peak value (*EQE*_up_=5% at 1.025 eV) is remarkably high considering that only sunlight in the range 1,000–1,060 nm can be received by the solar collector as a consequence of the bandgaps attainable using the InGaAs material system. To reach the high-efficiency regime shown in Fig. 2a, lower bandgap materials are needed, which allow the solar collector to absorb much more solar energy.

However, under laboratory conditions it is possible to probe different regimes of operation, which cannot be achieved with solar illumination. The red region in Fig. 2b shows the predicted *EQE*_up_ under simulated laser illumination at 1,064 nm with a high power density of 500 kW cm^−2^. Although laser illumination is not a practical configuration for solar energy conversion, the model provides a useful guide for experimental characterization and indicates an operating regime in which the up-conversion efficiency is very high (*EQE*_up_=80%). This thermodynamic result therefore motivates the design of our samples and experiments that follow. Also shown in Fig. 2b is the reduction in *EQE*_up_ when carrier cooling is included (the series of red lines). The up-conversion efficiency remains very high, even for realistic levels of carrier cooling, *Q*=30–300 W cm^−2^ K^−1^, providing further evidence that this structure is a good candidate for exploring the thermal up-conversion mechanism in more detail.

### Hot-electron temperature from Stokes spectroscopy

Figure 3a shows the thermally broadened photoluminescence (PL) spectrum of reference sample C (the PL heterostructure). This illustrates the very large thermal broadening that can be achieved in MQW structures when illuminated with a laser of higher energy than the bandgap (in this case an 808-nm diode laser was used). The corresponding carrier temperature is shown in Fig. 3b and has been extracted from the PL data by high-energy tail fitting with the equations (1) and (2):


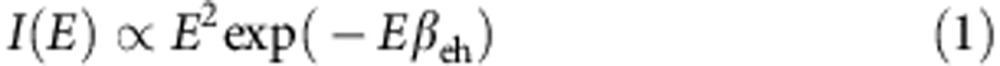


where *β*_eh_=(*k*_B_*T*_eh_)^−1^ in which *k*_B_ is the Boltzmann constant and *T*_eh_ is the hot electron and hole temperature. The horizontal error bars include uncertainty in the laser spot area and laser power, and the vertical error bars correspond to the uncertainty in the least squares fit. Figure 3b shows that (even under continuous-wave illumination) a remarkable hot-carrier temperature, slightly below 800 K, can be established in these structures. The temperature is limited by the transfer of energy from the electronic system to longitudinal optical (LO) phonons. A convenient metric that allows the relaxation rate for different materials and structures to be compared was introduced by LeBris *et al.*^11^. Writing the energy relaxation rate due to LO phonons as:


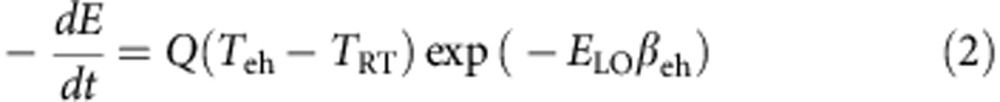


where *T*_RT_=292 K is the temperature of the lattice, *E*_LO_=36 meV is the zone-centre LO phonon energy and *Q* is a material-dependent constant called the cooling coefficient (W cm^−2^ K^−1^). In steady-state conditions the power lost to LO phonons must equal the power gained from the pump laser. The cooling coefficient can be extracted graphically by re-arranging the above equation in the form of *y*=*Qx*, and recording the gradient of the line shown in the inset of Fig. 3b. This gives a value of *Q*=30 W cm^−2^ *K*^−1^, which compares well with other values reported in the literature, which range from 2.5 to over 100 W cm^−2^ *K*^−1^ (refs 7, 8).

The inset also shows that subtracting two experimental values with similar magnitudes results in a large error, and this is the reason for the large relative errors when Δ*T* is close to zero. For large Δ*T* values the error also increases. This is because the high-energy tail fitting procedure begins to fail when the PL broadening is large. Moreover, the fitting procedure relies on the fact that the quantum well density of states is step like, thus the PL corresponds directly to the Boltzmann function in the fitting window (shown as the red lines on the PL in Fig. 3a). However, with very large Δ*T* values, the PL is broadened to such an extent that the PL in the fitting window is influenced by excitonic effects, thus breaking the main assumption of the tail-fitting procedure.

### Hot-electron temperature from anti-Stokes spectroscopy

Sample A, a solar cell (1,000 nm absorption edge) with an MQW-based solar collector (1,064 nm absorption edge), is illuminated with a 1,064-nm laser and the anti-Stokes PL is recorded. At this wavelength the solar cell is virtually transparent with absorptivity 1 × 10^−5^, resulting in the laser being strongly absorbed by the MQWs in the solar collector located below (absorptivity 0.1). The solar cell bias is held at short circuit by a source measurement unit. In this experimental configuration the solar cell emits neither PL or electroluminescence, allowing direct observation of the anti-Stokes PL emitted by the isolated MQWs.

Figure 4a shows the normalized anti-Stokes PL of sample A. As the laser power is increased, a clear broadening trend is observed. Note that the recorded PL is filtered by its passage through the solar cell, which suppresses features at shorter wavelengths (<1,020 nm), and by a notch filter (as indicated). It is also important to note that no wavelength shift is seen. A shift would indicate a change in bandgap caused by lattice heating. These data are clear evidence that hot carriers are being generated without a change in lattice temperature, and this is confirmed by high-energy tail fitting to extract the carrier temperature as shown in Fig. 4b, which shows a modest temperature gradient of Δ*T*=20 K.

### Thermophotonic device operation

When illuminated in the anti-Stokes configuration the power dependence of sample A's photocurrent can be used to extract additional information regarding the magnitude of the thermally up-converted photon flux emitted by the solar collector. Moreover, sample A should generate a photocurrent derived from three terms:


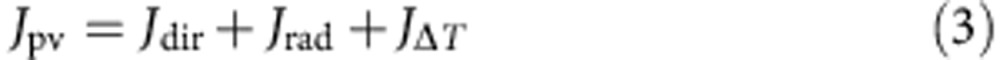


The small current generated by the 1,064-nm laser beam passing through the solar cell is given by *J*_dir_. Direct generation occurs because the laser can couple to states in the tail of the exciton absorption spectrum. It can be resolved in this experiment because of the high-intensity pump laser used and because lock-in amplification of photocurrent is extremely sensitive. The radiative coupling occurring between the MQW-based solar collector and the solar cell (in the absence of a temperature gradient) is denoted by *J*_rad_. Radiative coupling occurs because of natural broadening of the emission spectra at room temperature. In an ideal solar cell, *J*_dir_ and *J*_rad_ will scale linearly with laser intensity. The final component *J*_Δ*T*_ is the thermally up-converted photocurrent generated in the solar cell by the radiative recombination of hot carriers in the solar collector. Here *J*_Δ*T*_ is nonlinear with pump intensity (*I*) and is proportional to the PL broadening (*I*_PLB_). The broadening is caused by Auger processes that eject carriers to high-lying states in the bands, therefore *I*_PLB_∝*I*^3^. The solar cell has a linear response with light intensity so the Auger power dependence is reflected in the measured nonlinear component of the photocurrent *J*_Δ*T*_∝*I*_PLB_∝*I*^3^.

Figure 5a shows the photocurrent for sample A and reference sample B recorded as the laser power is swept over four orders of magnitude. Sample B allows direct measurements of *J*_dir_ because it does not have an MQW-based solar collector. By comparing the photocurrent of these two samples we infer that the highly linear response of sample A is due to the *J*_rad_ component and that *J*_rad_ is four orders of magnitude larger than directly induced photocurrent *J*_dir_.

Figure 5b,c shows a key result: that the nonlinear photocurrent region is correlated with the onset of the carrier temperature in the collector.

## Discussion

The anti-Stokes PL shown in Fig. 4 is drastically different to the Stokes PL shown in Fig. 3. In the Stokes case, hot carriers are generated directly by the absorption of a photon with energy much higher than the bandgap. Therefore, the power dependence versus temperature is related to how quickly the hot carriers lose energy via emission of LO phonons. In the anti-Stokes case, cool carriers are generated by the laser in the ground state and the power dependence versus temperature is related to how quickly the carriers are heated by Auger processes. At first this would appear to cause a problem: anti-Stokes illumination can refrigerate the material via radiative cooling if the total emitted power is larger than the power absorbed from the laser^10^. However, we observe the opposite.

We believe hot-carrier generation occurs in our samples as a result of entering a high photogenerated carrier density regime (we estimate between 10^18^ and 10^19^ cm^−3^) in which Auger recombination can begin to significantly heat the carrier population. In similar conditions, PL up-conversion has been observed before in nanostructures via Auger processes^12^, which supports the Auger-heating hypothesis.

Further analysis can be performed by fitting the nonlinear power dependence of the photocurrent. In fitting the power dependence we ignore the small *J*_dir_ contribution and use the following equation that corresponds to the black line in Fig. 5c:


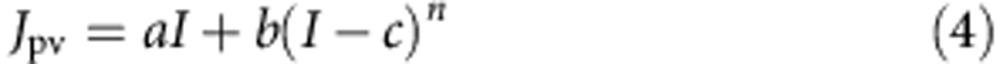


The blue line in Fig. 5c is a linear (*n*=1) fit accounting for the *J*_rad_=*aI* component of the overall current. A clear deviation from the linearity is seen at high illumination intensity, which is accounted for by the nonlinear term, *J*_Δ*T*_=*b*(*I*−*c*)^*n*^, which is constrained to be zero when *I*<*c* and non-zero after the threshold *c* has been reached. Equation (4) has two parameters that control the physics: *n* (the power) and *c* (the threshold for carrier heating), thus many pairs of *n* and *c* values could be used to fit the observed data. However, only a small subset of these have physical meaning: *n* must be between 1 and 3, and *c* must be close to the value observed in the spectroscopy measurements of Fig. 4b. To test the hypothesis that Auger recombination is driving the increase in carrier temperature we fix *n*=3 and use a least squares fit to extract the threshold from the data (justification for using *n*=3 appears in the methods section). By comparing the values we find remarkably good agreement within errors, the threshold value from spectroscopy is *c*=95±30 kW cm^−2^, while the fitting procedure recovers *c*=117±25 kW cm^−2^. The fitting procedure is phenomenological, yet the strong correlation supports the hypothesis of Auger heating in the MQW absorbers.

Figure 5d shows the photocurrent data and fit line converted to an up-conversion efficiency (more details can be found in the Methods section). The red region clearly shows the regime in which the solar cell behaves as a thermophotonic converter: the up-conversion efficiency is boosted from *EQE*_up_=2.5% (caused exclusively by radiative coupling) to *EQE*_up_=2.9% once a thermal gradient is established. Note the MQW layers in the solar cell and absorber have absorptivity *a*_up_=10% for a single pass. Thus, the internal up-conversion efficiency *IQE*_up_=*EQE*_up_/*a*_up_=29%, which is remarkably high. It is also in reasonable agreement with the *EQE*_up_ value predicted from the idealized thermodynamic model, which assumes unity absorptivity (Fig. 2b).

The key result is the first demonstration of a thermophotonic emitter coupled to a conventional solar cell. This has been shown through a series of spectroscopic and photocurrent measurements, which push the solar collector into a regime of efficient thermal up-conversion. Under laser illumination electron–hole pairs are generated in an MQW absorber, which establishes an electro-chemical potential gradient. As the pump intensity is increased, a thermal gradient is also established in the collector, this enhances the overall emission rate. The measurements performed show the enhancement of the emission rate is due to the dual nature (photovoltaic and thermal) of the emitter material.

The novel aspect of this work is that the thermal gradient is maintained by hot electrons in an otherwise room temperature absorber material; this was confirmed through Stokes and anti-Stokes spectroscopy and by a high-energy tail-fitting procedure. Under resonant excitation with the ground state hot carriers are generated through an Auger-heating process. However, in III–V materials Auger processes are inefficient and electronic cooling is very rapid, and for these reasons a very high illumination intensity, of at least 100 kW cm^−2^, was needed to maintain a modest thermal gradient of Δ*T*=20 K. Unlike conventional thermophotovoltaic approaches, where the collector is located externally, the hot-carrier approach allows the collector to be integrated directly into the solar cell without degrading the cell performance. The internal location enhances the radiative transfer rate by *n*^2^≈12 × , compared with an externally located emitter. Furthermore, our device uses the same growth techniques and has the same form factor and the same efficiency limit (around 60%) as conventional concentrating multijunction solar cells. Therefore, in principle, it could be used as a substitute for these devices, without the need for changes to existing infrastructure.

The device design presented here was chosen because it promised the clearest route to demonstrate and quantify the thermophotonic approach. However, it is unlikely to be the final iteration of the device concept. Lower bandgap materials with better up-conversion characteristics are needed. In the near-term optical cavities with high *Q*-factors can improve the absorption and concentration of light above the 46,200 × limit over small volumes and wavelength ranges. Furthermore, control of the optical density of states can also be used to tailor the emission spectrum, and this will enhance the efficiency of the device^5^. By considering these options it is very likely that thermal up-conversion can be observed at more realistic concentration levels over the next few years. A complementary and potentially more practical approach is thermal down-conversion using the single-source configuration. With the hot-carrier collector placed first, it receives the full (non-truncated) solar spectrum, enabling significantly higher thermal gradients to be established; beyond which can be achieved with Auger heating. The success of all hot-carrier approaches hinges on the ratio between the cooling rate and generation rate. In this context, provided that the cooling is slow enough, the opportunity exists for a number of interesting high-efficiency devices, from solar cells to novel spectral converters. As shown here, spectral converters have the advantage that they can augment conventional solar cells to boost their efficiency when used in combination with high optical concentration.

## Methods

### Photoluminescence microscope

A PL microscope was designed and built with the dual purpose of focusing a laser beam to a diffraction-limited spot on the sample surface, and also to collect PL from the illuminated area. The beam path of the PL microscope is shown in Fig. 6a. A laser beam is first mechanically chopped, attenuated by neutral density filters (ND1) and steered towards the 90:10 beam splitter (BS1) by beam-steering mirrors M1 and M2. The two mirrors allow axis-independent adjustments of the beam path such that it can propagate along the optical axis of all the optical components. The high reflectance ratio of BS1 was chosen to keep the laser power on the sample as high as possible. After reflection from BS1, the beam enters the microscope objective and is focused to a diffraction-limited spot on the sample surface. PL and reflected and scattered laser light are collected by the objective and collimated. The beam enters BS1 again, and the transmitted component (around 10%) is directed along the imaging path or detection path by a removable mirror (M3). The imaging path consists of a 150-mm plano-convex lens (L1), which focuses the collimated PL on a charge-coupled device to form an image (Fig. 6b). Along the detection path, the PL first passes through a focusing lens (L2) with f-number matched to the entrance of a single-grating monochromator. After the lens, a laser blocking filter (F1) is used to prevent laser light entering the detector. For the 808-nm laser, a low-pass (that is, long wavelength passing) filter is used, and for the 1,064-nm laser a holographic notch filter (HSPF-1064.0-1.0 with optical density of six from Kaiser Optical Systems) is used. At the exit of the monochromator, a calibrated Newport 818-SL (Ge) photodiode collects the monochromatic signal (D2).

The optical system also has built-in power monitoring of the laser beam. The component of the laser beam initially transmitted through the BS1 is attenuated by calibrated neutral density filters (ND2) and enters a calibrated silicon photodiode (D1). A calibrated two-channel transconductance amplifier with 10^6^ V A^−1^ voltage gain converts the photocurrent signal (on the order of nA) to a voltage signal, which is sent to the lock-in amplifier. All the optical components along the beam path between the entrance of the optical system and the photodiode surface have been calibrated for optical throughput and in this way, the beam power entering any of the components—including on the sample surface—is known.

The photocurrent signal recorded by the Ge photodiode (D2) is first amplified by the transconductance amplifier (using the second channel). The signal is then amplified further by the lock-in amplifier. Similarly, when a solar cell sample is being tested (for example, when measuring the photocurrent power dependence) electrical connections are passed to the transconductance amplifier first and then on to the lock-in amplifier for measurement.

Before entering the optical system the beam is first expanded. This ensures that the beam is as large as possible on the entrance aperture of the microscope objective, which in turn allows the objective to focus the laser to a small diffraction-limited spot. Two lasers were used: a 808-nm diode laser and 1,064-nm DPSS laser. The 808-nm beam is first collimated by an aspherical lens, then astigmatism correction is applied using a lens pair of cylindrical lenses, which expand the slow axis. This increases the beam area to fill the entrance aperture of the objective without clipping the beam. Although the beam shape is improved, it remains noticeably asymmetric with approximate dimensions of 4 × 1 mm at the entrance aperture of the microscope objective. The 1,064-nm DPSS laser generates a high-quality TEM00 Gaussian beam, and for this reason beam expansion is easily achieved by a pair of plano-convex lenses. The beam is expanded to a diameter of ∼5 mm and fills the entrance aperture of the objective.

As discussed above, the integrated power monitoring allows the power on the sample surface to be known. However, it is the power density (Wm^−2^) that is of fundamental importance for the physical concepts explored in this paper. Therefore, accurate knowledge of the laser spot size on the sample surface is also required. To measure this, a 50-μm slit is placed at the focal point of the microscope and an image of the slit is taken with back illumination (see Fig. 6b, the image marked ‘Scale reference'). This provides a magnification parameter for the optical system with units of pixels μm^−1^. An image of the laser spot on the sample surface is then taken and converted to absolute units using the magnification parameter (Fig. 6b). In this way, the area of the beam can be determined and the absolute beam irradiance calculated. Figure 6c shows a cross-section of the 1,064-nm laser spot intensity shown in Fig. 6b. For the 1,064-nm beam, this procedure yields a 1/*e*^2^=4 μm beam diameter, which corresponds to a laser spot size of around 12.5 μm^2^.

### Sample descriptions

**Figure 1:**
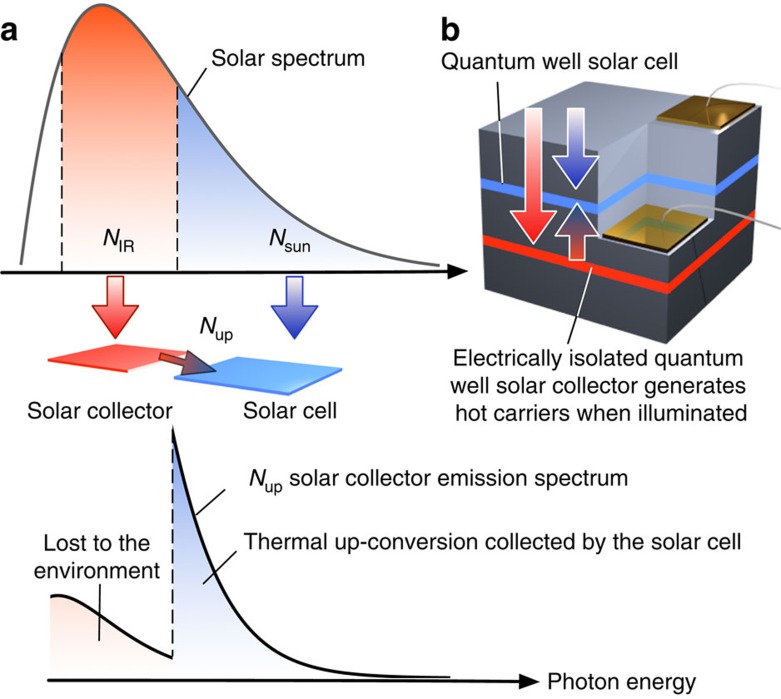
Thermal up-conversion with hot carriers: concept and implementation. (**a**) Energy flow showing solar photons entering the solar cell (*N*_sun_) and sub-bandgap photons (*N*_IR_) entering the solar collector. The solar collector generates hot carriers and emits broadened PL (*N*_up_) towards the solar cell. The additional flux of above-gap photons, which have been thermally up-converted in the solar collector, boost the efficiency of the solar cell. The thermally up-converted component is enhanced by factor *n*^2^≈12 × , compared with the component that is lost to the environment, because it propagates in the internal optical modes of the semiconductor. (**b**) The structure of sample A used to demonstrate the efficiency enhancement due to thermal up-conversion. The device consists of a InGaAs/GaAsP quantum well solar cell (with absorption edge 1,000 nm) with a rear contact and electrical isolation layer of GaAs. Below the solar cell, electrically isolated InGaAs/GaAsP quantum wells (with absorption edge 1,060 nm) are located. This layer acts as a solar collector and emits thermally broadened PL to the solar cell.

**Figure 2:**
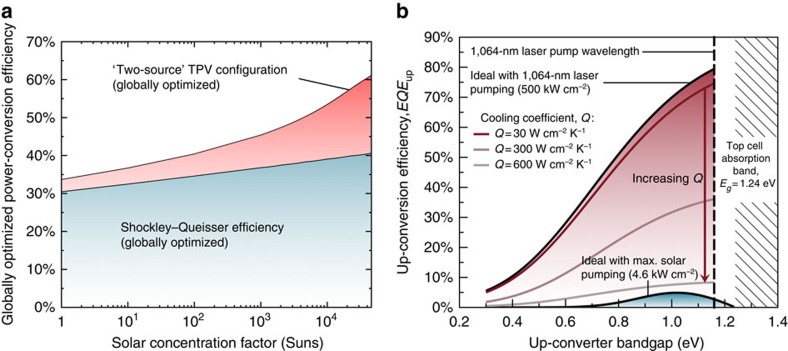
Thermodynamic power conversion and optical thermal up-conversion efficiency (*EQE*_up_) limits for the two-source configuration. (**a**) Globally optimized thermodynamic power-conversion efficiency limit for the two-source configuration (red region) compared with the Shockley–Queisser efficiency as a function of the solar concentration factor. (**b**) Thermal up-conversion efficiency under solar (blue region) and high-intensity laser (red region) illumination for a fixed solar cell bandgap (1,000 nm) as a function of the solar collector bandgap. The red lines indicate the reduction in *EQE*_up_ as cooling coefficient *Q* increases. All simulations are performed in the far-field limit with the exchange of fluxes occurring inside the semiconductor with refractive index 3.5 and assuming the top solar cell has bandgap *E*_g_=1.24 eV.

**Figure 3:**
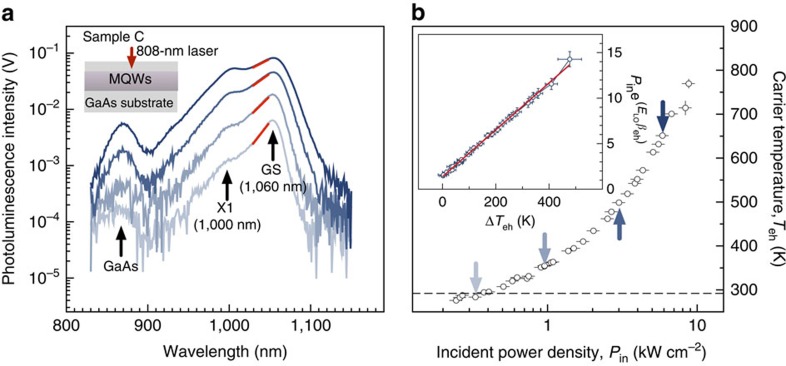
Stokes PL and extracted carrier temperatures for a heterostructure (sample C) containing 1,060** **nm absorption threshold MQWs. (**a**) Stokes PL generated by an 808-nm pump laser with irradiance 0.33, 0.95, 3.00 and 5.00 kW cm^−2^. The red lines show the high-energy tail-fitting locations. The labelled emission peaks correspond to the GaAs capping layer, the MQW excited state (X1) and ground state (GS). (**b**) Electron–hole temperature (*T*_eh_) versus laser irradiance (*P*_in_). Carrier temperature has been extracted from high-energy tail fitting. The arrows indicate the data points corresponding to the spectra shown in **a**. The inset shows the graphical process for extracting the cooling coefficient of the hot-carrier ensemble by rearranging equation (2). The gradient corresponds to the cooling coefficient of *Q*=30 W cm^−2^ K^−1^.

**Figure 4:**
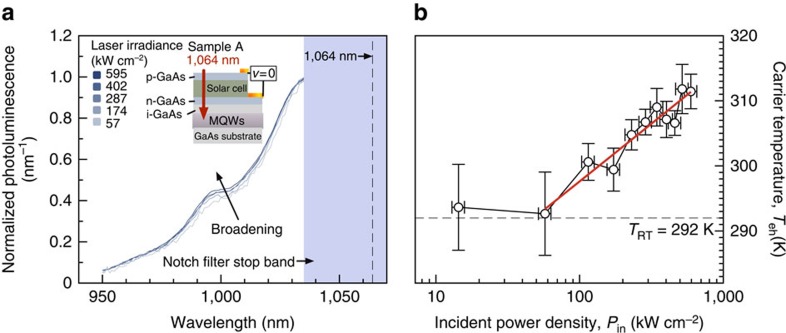
Anti-Stokes PL and extracted carrier temperatures for a full solar cell structure with an MQW solar collector (sample A) with absorption threshold 1,060 nm. (**a**) Anti-Stokes PL of sample A under 1,064-nm laser illumination. The laser is strongly absorbed in the isolated MQWs, which have absorptivity *a*=0.1. At this wavelength the absorptivity of the solar cell is very small (*a*=1 × 10^−5^). The broadening indicates the generation of hot carriers. The inset shows the sample and experimental conditions used in taking the measurements. The solar cell is biased at short circuit. (**b**) Electron–hole temperature (*T*_eh_) versus laser irradiance (*P*_in_) showing the threshold for carrier heating at between 50 and 125 kW cm^−2^.

We have designed and grown three samples. Sample A (shown schematically in Fig. 1b) is a full structure containing a solar cell with a 1,000 nm absorption threshold and a solar collector layer grown behind the solar cell with a longer wavelength absorption threshold of 1,060 nm. In_0.195_GaAs/GaAsP_0.32_ MQWs with a well/half-barrier thickness of 6.3/7.4 nm are used in the i-region of the solar cell to adjust the absorption threshold to 1,000 nm. Wider quantum wells with slightly more indium are used for the solar collector layer: In_0.255_GaAs/GaAsP_0.29_ MQWs with a well/half-barrier thickness of 15/10 nm. Details of the full structure is shown in Table 1. Samples B and C are control samples with descriptions in Tables 2 and 3, respectively. Sample B is a solar cell reference sample without the solar collector region and is used to characterize the level of directly generated photocurrent by the 1,064-nm laser. Sample C is a PL heterostructure containing only the 1,060-nm threshold quantum wells, it was used to perform the Stokes spectroscopy, which established the rate of hot-carrier cooling.

### Sample growth and processing

All samples were prepared by a planetary metal-organic vapour phase epitaxy (MOVPE) reactor (AIXTRON HT2000) on *n*-doped GaAs (100) substrates. Trimethylgallium, trimethylindium, tertiarybutylarsine and tertiarybutylphosphine were used as metal-organic sources for Ga, In, As and P, respectively. The reactor temperature and pressure during growth were nominally 610 °C and 100 mbar, respectively. The V/III ratio and growth rate were, respectively, 15 and 10 nm min^−1^; except for n-GaAs contact and base layers where the values of 10 and 40 nm min^−1^ were used. *In situ* wafer curvature measurement (Epicurve from LayTec) was used to monitor strain accumulation during growth and optimize the wafer bowing of the InGaAs and GaAsP regions for zero net strain. After MOVPE growth, the high-resolution X-ray diffraction (004) scan was used to confirm the thickness and atomic composition of the wells and barriers.

The device processing of sample A requires the formation of a top surface contact that is electrically connected to the rear of the solar cell. This is required to electrically isolate the up-converting MQW layer grown directly below the solar cell. The procedure for exposing the rear contact layer (n-GaAs) follows an involved multi-step process. First, a photoresist mask was deposited then photolithography was used to create a metal grid pattern. A Ti/Au (20/250 nm) contact was then deposited using an electron beam evaporator followed by lifting-off of the photoresist. Next, a 1-μm thick layer of SiO_2_ was sputtered as an etching mask. Photolithography and chemical etching were used to pattern square areas on the SiO_2_ mask followed by inductively coupled plasma dry etching of the n-GaAs contact layer. An AuGe/Ni (350/25 nm) metal contact was deposited by a thermal evaporator using a lithography lift-off technique. This contact was annealed at 380 °C for 5 min by a rapid thermal annealing system. The SiO_2_ mask was then removed by buffered hydrofluoric acid solution. Last, the p-GaAs contact layer (located under the Ti/Au metal) was protected from a H_3_PO_4_:H_2_O_2_ etch solution whilst other areas were etched away.

### Power dependence of Auger-driven thermal up-conversion

Auger recombination is a three-particle process, therefore its rate scales with the cube of electron (*n*) and (*p*) hole density. More specifically:


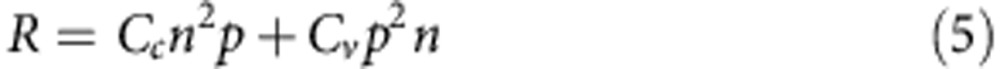


where the first and second terms account for the Auger processes, which leave a hot electron in the conduction band and a hot hole in the valence band, respectively.

In the samples considered here, the IR laser of irradiance *I* generates carriers directly into the ground state of the quantum wells, therefore:


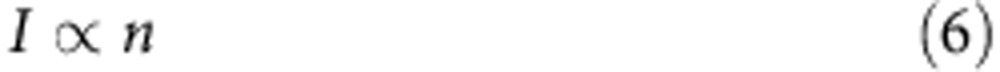



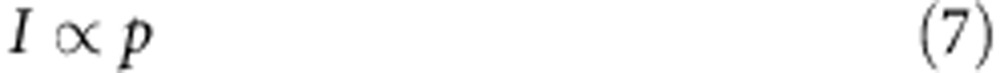


Substituting this into the rate equations (5) yields:


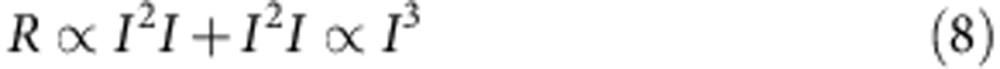


In intrinsic semiconductors, such as our samples, under high illumination intensity we should therefore expect *R*∝*I*^3^ dependence. This is because the free carrier population contributed by *n*-type background doping *n*=*N*_d_=10^14^–10^15^ cm^−3^ is insignificant compared with the photogenerated contribution *n*=*p*=10^19^–10^20^ cm^−3^.

However, a different Auger rate could be expected for *n*-doped samples at weaker illumination intensities, where the photogenerated contribution is *n*+*p*<*N*_d_. In such a case, the Auger rate is limited by the available number of photogenerated holes. Therefore:


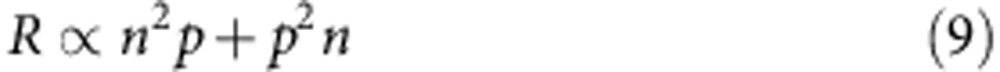



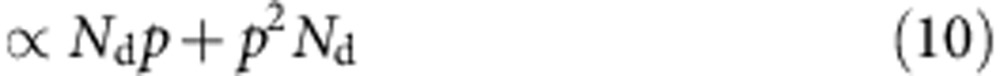



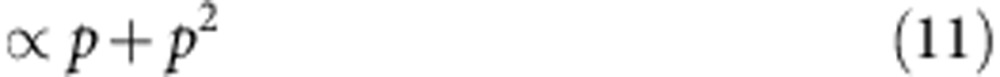


Again using equations (6) we find that at low illumination intensity:


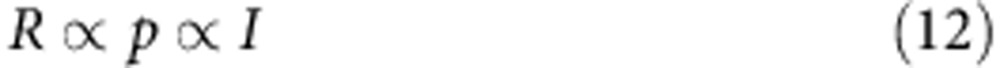


and at high illumination intensity:


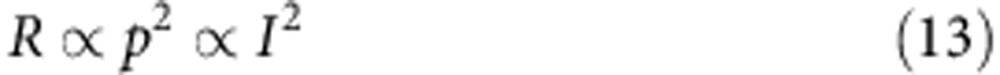


Therefore, the order of the Auger process is complex and depends on the doping condition of the sample and the illumination intensity used. The Auger rate can in principle range from *n*=1 to 3 power dependence. However, for the samples and experimental conditions discussed here (that is, intrinsic semiconductors under very intense illumination) we expect to be firmly in the *n*=3 regime, which is the justification for using the *R*∝*I*^3^ assumption in the fitting procedure.

### External and internal up-conversion efficiency

The external up-conversion efficiency is defined as the ratio of the up-converted photon flux (photons per second) received by the solar cell to the infrared photon flux (from the laser), which enters the quantum wells:


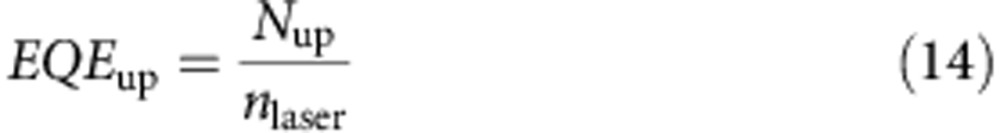


The laser irradiance *I*, spot area *A* and energy per photon *ℏω* is accurately known, allowing the *n*_laser_ to be calculated as:


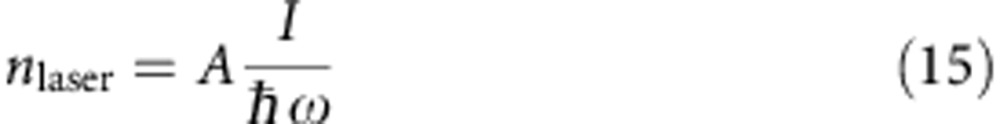


The photocurrent measurement can be used to find the up-converted photon flux entering the solar cell:


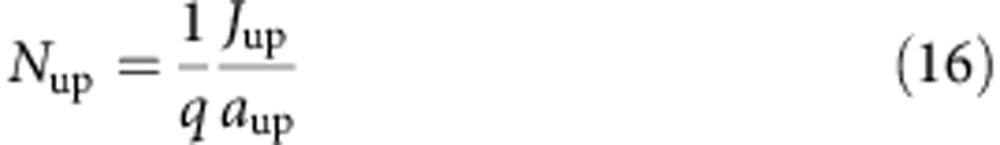


where *q* is the electronic charge and *a*_up_(1,000 nm)=10% is the absorptivity of the top cell.

**Figure 5:**
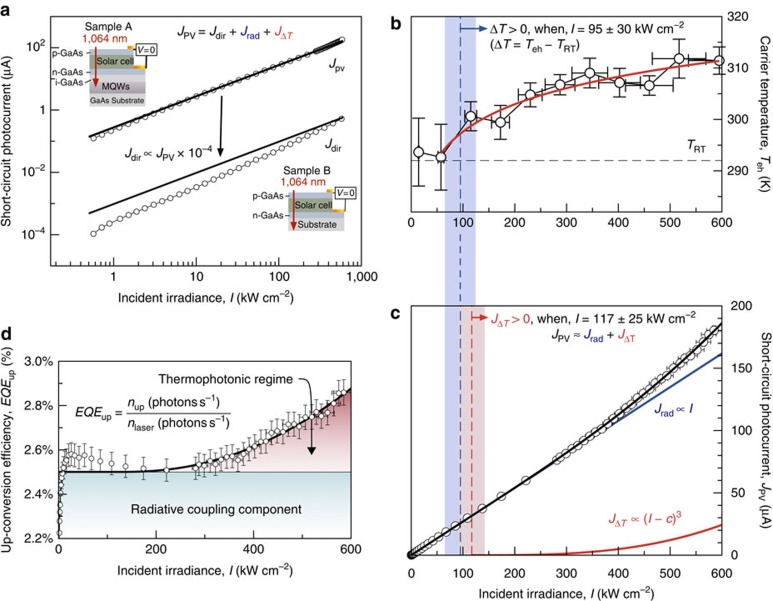
Photocurrent power dependence under anti-Stokes illumination. (**a**) The power dependence of sample A, in which *J*_pv_ is comprised of three components: radiative coupling *J*_rad_, direct generation *J*_dir_ and emission enhancement due to entering the thermophotonic regime *J*_Δ*T*_. The inset diagrams show the sample structure and experimental conditions for the measurements. Sample A is a solar cell with an MQW-based solar collector and sample B is an identical reference solar cell without a solar collector. The lines are linear fits to experimental data showing that the solar cell photocurrent is mainly due to radiative coupling between the solar collector and the solar cell. The data also show that the direct generation component is negligible being four orders of magnitude smaller than other terms. (**b**) The extracted carrier temperature from anti-Stokes PL shows the onset of carrier heating at 95±30 kW cm^−2^. The horizontal error bars represent the measurement error in laser intensity and spot size, the vertical errors bars correspond to the standard error associated with the least squares fitting. (**c**) Fitted power-dependent photocurrent of sample A (black) with linear *J*_rad_ (blue) and nonlinear *J*_Δ*T*_ (red) components. Note that *J*_dir_ has been ignored because it contributes negligibly to the overall photocurrent. The data show an onset of carrier heating of 117±25 kW cm^−2^, which is in good agreement with the value extracted from PL. Horizontal error bars have the same meaning as in **b** and vertical error bars are the standard error in the photocurrent measurement. (**d**) Experimentally determined up-conversion efficiency *EQE*_up_, which is the ratio of photons per second up-converted to *n*_laser_, the number of entering photons per second from the laser. The thermophotonic regime is indicated in which the optical energy transferred to the solar cell is enhanced due to the solar collector having both a chemical potential and a thermal gradient. The errors bars are calculated by propagating the standard error values from photocurrent data shown in **c**.

**Figure 6:**
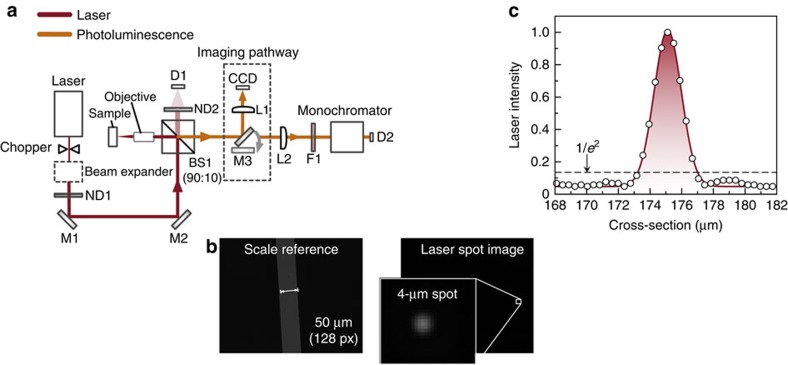
PL microscope for high-intensity illumination and measurement of laser spot size. (**a**) The beam paths for the illumination laser and PL are shown in red and orange, respectively. The text has a full description of the all optical components. (**b**) Images taken of the reference slit of known size, which enables the magnification of the optical system to be characterized in terms of pixels (px) μm^−1^. The slits are exchanged for a semiconductor device and the laser spot is imaged on the surface. (**c**) Intensity profile cross-section of the laser spot image showing diffraction-limited performance. The size of the laser spot is determined by taking the 1/*e*^2^ diameter of the laser spot.

In this way we calculate a peak up-conversion efficiency of around 3%, as shown in Fig. 5d.

In this article the lower multiple quantum wells have absorptivity *a*_IR_(1,064 nm)=10% at the laser wavelength. Thus, the internal up-conversion efficiency gives an upper limit of


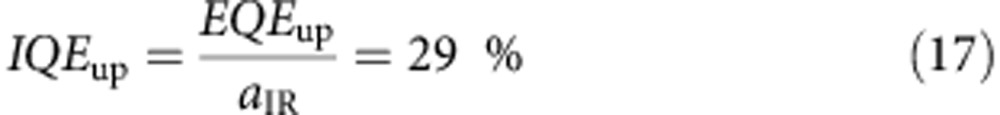


which is remarkably high.

## Additional information

**How to cite this article:** Farrell, D. J. *et al.* A hot-electron thermophotonic solar cell demonstrated by thermal up-conversion of sub-bandgap photons. *Nat. Commun.* 6:8685 doi: 10.1038/ncomms9685 (2015).

## Figures and Tables

**Table 1:** Sample A device structure: full structure with a top MQW solar cell of absorption threshold 1,000 nm and a bottom MQW-based solar collector with an absorption threshold of 1,060 nm.

**Layer**	**Composition**		
	**Material**	**Thickness (nm)**	
Front grid	Ti/Au	250	
p-Contact	p-GaAs, 1 × 10^19^ cm^−3^	20	
Window	p-InGaAs, 5 × 10^18^ cm^−3^	30	
Emitter	p-GaAs, 1 × 10^18^ cm^−3^	200	
Spacer	i-GaAs	120	
Barrier	i-GaAsP_0.32_	7.4	
Well	i-In_0.195_GaAs	6.3	20 × MQWs
Barrier	i-GaAsP_0.32_	7.4	
Spacer	i-GaAs	120	
Base	n-GaAs, 5 × 10^17^ cm^−3^	1,000	
n-Contact	n-GaAs, 2 × 10^18^ cm^−3^	1,000	
Depletion	i-GaAs	240	
Barrier	i-GaAsP_0.29_	10	
Well	i-In_0.255_GaAs	15	20 × MQWs
Barrier	i-GaAsP_0.29_	10	
BSF	i-InGaP	30	
Buffer	i-GaAs	80	
Substrate	GaAs	—	

**Table 2:** Sample B structure: reference solar cell with a MQW absorption threshold of 1,000 nm. This device does not contain a solar collector layer.

**Layer**	**Composition**		
	**Material**	**Thickness (nm)**	
Front grid	Ti/Au	250	
p-Contact	p-GaAs, 1 × 10^19^ cm^−3^	20	
Window	p-InGaAs, 5 × 10^18^ cm^−3^	30	
Emitter	p-GaAs, 1 × 10^18^ cm^−3^	200	
Spacer	i-GaAs	120	
Barrier	i-GaAsP_0.32_	7.4	
Well	i-In_0.195_GaAs	6.3	20 × MQWs
Barrier	i-GaAsP_0.32_	7.4	
Spacer	i-GaAs	120	
Base	n-GaAs, 5 × 10^17^ cm^−3^	1,000	
n-Contact	n-GaAs, 2 × 10^18^ cm^−3^	1,000	
Back contact	Ti/Au	250	
Substrate	GaAs	—	

**Table 3:** Sample C structure: PL heterostructure containing only the 1,060-nm absorption threshold MQWs.

**Layer**	**Composition**		
	**Material**	**Thickness (nm)**	
Cap	i-GaAs	250	
Barrier	i-GaAsP 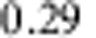	10	
Well	i-In 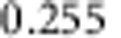 GaAs	15	10 × MQWs
Barrier	i-GaAsP 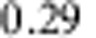	10	
Buffer	i-GaAs	50	
Substrate	GaAs	—	
